# 1-(2-Meth­oxy­phen­yl)-2-{[2-(2-meth­oxy­phen­yl)hydrazinyl­idene](nitro)­meth­yl}diazene

**DOI:** 10.1107/S1600536812004175

**Published:** 2012-02-04

**Authors:** Karel G. von Eschwege, Fabian Muller, Tania N. Hill

**Affiliations:** a Department of Chemistry, University of the Free State, PO Box 339, Bloemfontein 9300, South Africa

## Abstract

In the title compound, C_15_H_15_N_5_O_4_, a nitro­formazan derivative, the formazan unit is essentially planar with an r.m.s. deviation of 0.0204 (6) Å and adopts a closed *syn*,*s-cis* configuration with an intra­molecular N—H⋯N hydrogen bond. The formazan plane makes dihedral angles of 4.32 (5) and 24.35 (5)° with the benzene rings. The dihedral angle between the formazan plane and the nitro group is 12.58 (8)°. In the crystal, C—H⋯O inter­actions connect the mol­ecules into an inversion dimer.

## Related literature

For synthetic background, see: Pelkis *et al.* (1957 ▶). For applications of formazans, see: Irving (1977 ▶). For related structures, see: Gilroy *et al.* (2008 ▶); Laing (1977 ▶); Mito *et al.* (1997 ▶); von Eschwege *et al.* (2011 ▶, 2012 ▶); von Eschwege & Swarts (2010 ▶).
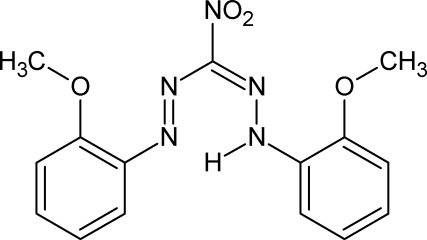


## Experimental

### 

#### Crystal data

C_15_H_15_N_5_O_4_
*M* *_r_* = 329.32
Triclinic, 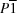

*a* = 7.2025 (5) Å
*b* = 10.9574 (8) Å
*c* = 11.2190 (9) Å
α = 117.188 (2)°
β = 91.416 (2)°
γ = 107.251 (2)°
*V* = 738.66 (10) Å^3^
*Z* = 2
Mo *K*α radiation
μ = 0.11 mm^−1^
*T* = 100 K
0.25 × 0.21 × 0.06 mm

#### Data collection

Bruker X8 APEXII 4K KappaCCD diffractometer
Absorption correction: multi-scan (*SADABS*; Bruker, 2004 ▶) *T*_min_ = 0.668, *T*_max_ = 0.746
10250 measured reflections
3431 independent reflections
2824 reflections with *I* > 2σ(*I*)
*R*_int_ = 0.027

#### Refinement

*R*[*F* ^2^ > 2σ(*F* ^2^)] = 0.037
*wR*(*F* ^2^) = 0.11
*S* = 1.04
3431 reflections
223 parameters
H atoms treated by a mixture of independent and constrained refinement
Δρ_max_ = 0.32 e Å^−3^
Δρ_min_ = −0.22 e Å^−3^

### 

Data collection: *APEX2* (Bruker, 2005 ▶); cell refinement: *SAINT-Plus* (Bruker, 2004 ▶); data reduction: *SAINT-Plus*; program(s) used to solve structure: *SHELXS97* (Sheldrick, 2008 ▶); program(s) used to refine structure: *SHELXL97* (Sheldrick, 2008 ▶); molecular graphics: *DIAMOND* (Brandenburg & Putz, 2005 ▶); software used to prepare material for publication: *WinGX* (Farrugia, 1999 ▶).

## Supplementary Material

Crystal structure: contains datablock(s) global, I. DOI: 10.1107/S1600536812004175/is5057sup1.cif

Structure factors: contains datablock(s) I. DOI: 10.1107/S1600536812004175/is5057Isup2.hkl

Supplementary material file. DOI: 10.1107/S1600536812004175/is5057Isup3.cml

Additional supplementary materials:  crystallographic information; 3D view; checkCIF report

## Figures and Tables

**Table 1:** Hydrogen-bond geometry (Å, °)

*D*—H⋯*A*	*D*—H	H⋯*A*	*D*⋯*A*	*D*—H⋯*A*
N4—H4⋯N2	1.10 (3)	1.73 (3)	2.6117 (15)	134 (3)
C17—H17*B*⋯O1^i^	0.98	2.47	3.3325 (16)	146
C27—H27*C*⋯O2^i^	0.98	2.65	3.3907 (17)	133

Symmetry code: (i) 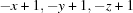.

**Fig. 1.**
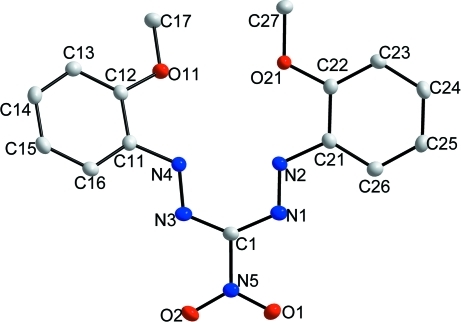
The molecular structure of the title compound with 50% probability displacement ellipsoids for non-H atoms.

**Fig. 2.**
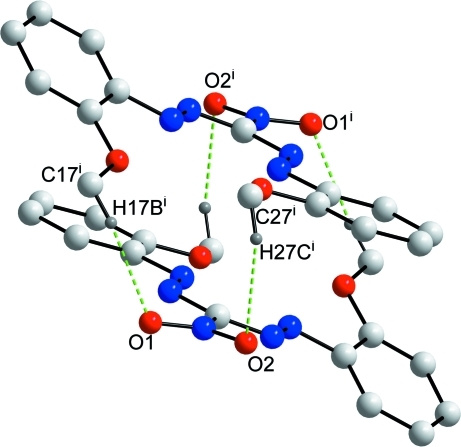
Intermolecular hydrogen bond interactions (dashed bonds) of the title compound. Non-relevant H atoms have been omitted for clarity. [Symmetry code: (i) 1 - x, 1 - y, 1 - z.]

**Fig. 3.**
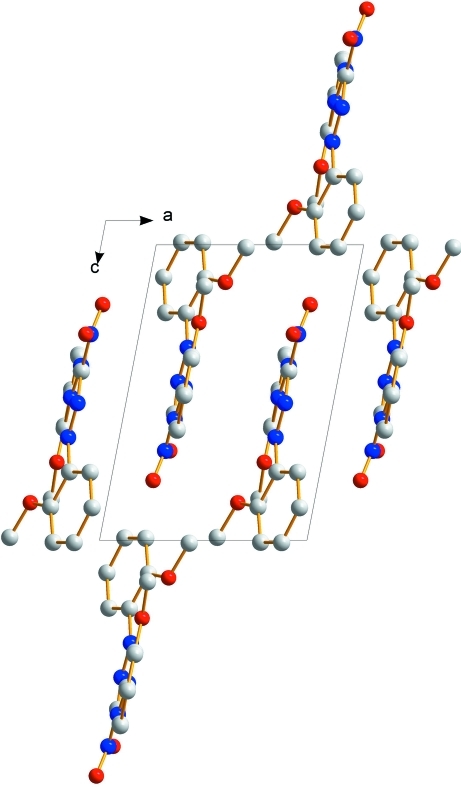
A packing diagram of the title compound, illustrating the parallel sheet configuration as viewed along the b axis.

## supplementary crystallographic information

### Comment

During synthesis of the versatile trace metal analysis dithizone reagent, aniline is first diazotized and then treated with nitromethane to form the bright orange–red nitroformazan product (Pelkis *et al.*, 1957). Ammonia and hydrogen sulfide gas is used to substitute the nitro group with sulfur towards the formation of dithizone, the chemistry of which is extensively described by Irving (1977). Single crystal X-ray structures of nitroformazan derivatives were determined by Gilroy *et al.* (2008) and Mito *et al.* (1997), and the dithizone structure by Laing (1977), while we performed extensive DFT (von Eschwege *et al.*, 2011) and electrochemistry studies (von Eschwege & Swarts, 2010) on the free ligand. We recently embarked on a study during which we synthesized a series of electronically altered dithizones for the purpose of investigating its altered redox and structural properties. During this process orange 1,5-bis(2-methoxyphenyl)-3-nitroformazan (I, Fig. 1) crystals suitable for X-ray crystallography were grown from an acetone solution overlaid with n-hexane.

The formazan backbone (N4—N3—C1—N1—N2) was found to be highly delocalized, showing minimal bond-length alternation [N1—N2 1.290 (1), N3—N4 1.301 (1), N1—C1 1.356 (2), N3—C1 1.325 (2) Å] which are similar to the values found by Gilroy *et al.* (2008) for the `closed' nitroformazan derivatives. The backbone was found to be essentially planar with an r.m.s. deviation of 0.0204 Å, and with a maximum deviation 0.0297 (8) Å for atom C1. The N—H bond in the expected intramolecular N—H···N hydrogen bond is elongated due to bridging with a distance of 1.10 (3) Å. Two additional hydrogen bond interactions (C17—H17B···O1^i^ and C27—H27C···O2^i^; Table 1) are observed between the O atoms of the nitro substituent on the formazan backbone with an adjacent molecule, resulting in the formation of 'dimeric' unit (Fig. 2) and a parallel sheet configuration when viewed along the *b*-axis (Fig. 3). The nitro group is twisted by 12.58 (8)° relative to the plane of the formazan backbone, while average twisting is observed for the phenyl substituents [4.32 (5)° and 24.35 (5)°].

### Experimental

Solvents (AR) purchased from Merck and reagents from Sigma–Aldrich were used without further purification. The *ortho*-methoxy derivative of nitroformazan was prepared according to the procedure reported by Pelkis *et al.* (1957). M.p. 164 °C; λ_max_ (dicloromethane) 362, 462 nm. δH (600 MHz, CDCl_3_) 14.92 (1*H*, s, NH), 4.03 (6*H*, s, OCH_3_), 8.00–7.04 (8*H*, m, C_6_H_4_).

### Refinement

The C-bound H atoms were placed in geometrically idealized positions (C—H = 0.95 or 0.98 Å) and constrained to ride on their parent atoms. The imine H atom (H4) bonded to the dithizone group was located in a difference Fourier map and refined freely.

### Crystal data

**Table:**

C_15_H_15_N_5_O_4_	*Z* = 2
*M**_r_* = 329.32	*F*(000) = 344
Triclinic, *P*1	*D*_x_ = 1.481 Mg m^−^^3^
Hall symbol: -P 1	Mo *K*α radiation, λ = 0.71073 Å
*a* = 7.2025 (5) Å	Cell parameters from 5015 reflections
*b* = 10.9574 (8) Å	θ = 3.0–28.4°
*c* = 11.2190 (9) Å	µ = 0.11 mm^−^^1^
α = 117.188 (2)°	*T* = 100 K
β = 91.416 (2)°	Cuboid, red
γ = 107.251 (2)°	0.25 × 0.21 × 0.06 mm
*V* = 738.66 (10) Å^3^	

### Data collection

**Table:**

Bruker X8 APEXII 4K KappaCCD diffractometer	3431 independent reflections
Radiation source: sealed tube	2824 reflections with *I* > 2σ(*I*)
Graphite monochromator	*R*_int_ = 0.027
Detector resolution: 512 pixels mm^-1^	θ_max_ = 28.0°, θ_min_ = 2.1°
φ and ω scans	*h* = −9→7
Absorption correction: multi-scan (*SADABS*; Bruker, 2004)	*k* = −10→14
*T*_min_ = 0.668, *T*_max_ = 0.746	*l* = −14→14
10250 measured reflections	

### Refinement

**Table:**

Refinement on *F*^2^	0 restraints
Least-squares matrix: full	H atoms treated by a mixture of independent and constrained refinement
*R*[*F*^2^ > 2σ(*F*^2^)] = 0.037	*w* = 1/[σ^2^(*F*_o_^2^) + (0.0567*P*)^2^ + 0.2301*P*] where *P* = (*F*_o_^2^ + 2*F*_c_^2^)/3
*wR*(*F*^2^) = 0.11	(Δ/σ)_max_ < 0.001
*S* = 1.04	Δρ_max_ = 0.32 e Å^−^^3^
3431 reflections	Δρ_min_ = −0.22 e Å^−^^3^
223 parameters	

### Special details

**Table:**

Geometry. All e.s.d.'s (except the e.s.d. in the dihedral angle between two l.s. planes) are estimated using the full covariance matrix. The cell e.s.d.'s are taken into account individually in the estimation of e.s.d.'s in distances, angles and torsion angles; correlations between e.s.d.'s in cell parameters are only used when they are defined by crystal symmetry. An approximate (isotropic) treatment of cell e.s.d.'s is used for estimating e.s.d.'s involving l.s. planes.

### Fractional atomic coordinates and isotropic or equivalent isotropic displacement parameters (Å^2^)

**Table:**

	*x*	*y*	*z*	*U*_iso_*/*U*_eq_	
C1	0.24373 (18)	0.35944 (13)	0.57201 (13)	0.0165 (3)	
C11	0.20805 (18)	0.09665 (13)	0.23162 (13)	0.0163 (3)	
C12	0.26683 (17)	0.09557 (13)	0.11311 (13)	0.0163 (3)	
C13	0.22134 (18)	−0.03694 (14)	−0.00672 (13)	0.0187 (3)	
H13	0.2594	−0.0385	−0.0877	0.022*	
C14	0.11980 (19)	−0.16760 (14)	−0.00785 (14)	0.0201 (3)	
H14	0.0874	−0.2579	−0.0901	0.024*	
C15	0.06606 (18)	−0.16640 (13)	0.11022 (14)	0.0193 (3)	
H15	0.0005	−0.2559	0.1095	0.023*	
C16	0.10804 (18)	−0.03447 (13)	0.22960 (13)	0.0175 (3)	
H16	0.0685	−0.0336	0.3101	0.021*	
C17	0.4352 (2)	0.23340 (14)	0.00981 (14)	0.0202 (3)	
H17A	0.5108	0.1675	−0.0269	0.03*	
H17B	0.5198	0.3334	0.0357	0.03*	
H17C	0.321	0.2022	−0.0598	0.03*	
C21	0.26964 (17)	0.64157 (13)	0.49771 (13)	0.0161 (3)	
C22	0.27401 (18)	0.65519 (13)	0.37900 (13)	0.0166 (3)	
C23	0.27496 (19)	0.78593 (14)	0.38634 (13)	0.0188 (3)	
H23	0.2767	0.796	0.3066	0.023*	
C24	0.27338 (19)	0.90125 (13)	0.50974 (14)	0.0203 (3)	
H24	0.2731	0.9897	0.5139	0.024*	
C25	0.2722 (2)	0.88841 (14)	0.62726 (14)	0.0215 (3)	
H25	0.2732	0.9684	0.7116	0.026*	
C26	0.26962 (19)	0.75873 (14)	0.62146 (13)	0.0192 (3)	
H26	0.2678	0.7497	0.7017	0.023*	
C27	0.2702 (2)	0.54524 (15)	0.14037 (13)	0.0226 (3)	
H27A	0.1481	0.5611	0.1218	0.034*	
H27B	0.2725	0.4537	0.0645	0.034*	
H27C	0.3848	0.6268	0.1502	0.034*	
N1	0.24998 (15)	0.49180 (11)	0.59013 (11)	0.0178 (2)	
N2	0.26924 (15)	0.50650 (11)	0.48294 (11)	0.0164 (2)	
N3	0.23306 (15)	0.23476 (11)	0.46437 (11)	0.0174 (2)	
N4	0.24656 (15)	0.23359 (11)	0.34850 (11)	0.0168 (2)	
N5	0.23346 (15)	0.34848 (11)	0.69742 (11)	0.0178 (2)	
O1	0.20306 (15)	0.44731 (10)	0.79751 (10)	0.0243 (2)	
O2	0.25603 (15)	0.24155 (10)	0.69729 (10)	0.0246 (2)	
O11	0.36796 (14)	0.22919 (9)	0.12699 (9)	0.0208 (2)	
O21	0.27761 (14)	0.53657 (9)	0.26384 (9)	0.0210 (2)	
H4	0.272 (5)	0.341 (4)	0.356 (3)	0.127 (12)*	

### Atomic displacement parameters (Å^2^)

**Table:**

	*U*^11^	*U*^22^	*U*^33^	*U*^12^	*U*^13^	*U*^23^
C1	0.0164 (6)	0.0176 (6)	0.0159 (6)	0.0044 (5)	0.0028 (4)	0.0094 (5)
C11	0.0154 (5)	0.0162 (5)	0.0161 (6)	0.0064 (5)	0.0006 (4)	0.0064 (5)
C12	0.0155 (5)	0.0164 (5)	0.0186 (6)	0.0064 (5)	0.0023 (5)	0.0093 (5)
C13	0.0199 (6)	0.0209 (6)	0.0168 (6)	0.0089 (5)	0.0039 (5)	0.0094 (5)
C14	0.0209 (6)	0.0166 (6)	0.0201 (7)	0.0078 (5)	0.0012 (5)	0.0060 (5)
C15	0.0192 (6)	0.0164 (6)	0.0240 (7)	0.0061 (5)	0.0013 (5)	0.0112 (5)
C16	0.0174 (6)	0.0201 (6)	0.0185 (6)	0.0076 (5)	0.0031 (5)	0.0115 (5)
C17	0.0219 (6)	0.0218 (6)	0.0204 (7)	0.0074 (5)	0.0069 (5)	0.0129 (5)
C21	0.0147 (5)	0.0156 (5)	0.0190 (6)	0.0051 (5)	0.0032 (5)	0.0092 (5)
C22	0.0159 (5)	0.0154 (5)	0.0169 (6)	0.0050 (5)	0.0027 (4)	0.0069 (5)
C23	0.0215 (6)	0.0190 (6)	0.0180 (6)	0.0071 (5)	0.0034 (5)	0.0108 (5)
C24	0.0223 (6)	0.0153 (6)	0.0236 (7)	0.0074 (5)	0.0022 (5)	0.0092 (5)
C25	0.0261 (6)	0.0176 (6)	0.0183 (6)	0.0098 (5)	0.0044 (5)	0.0054 (5)
C26	0.0215 (6)	0.0208 (6)	0.0167 (6)	0.0084 (5)	0.0048 (5)	0.0094 (5)
C27	0.0320 (7)	0.0222 (6)	0.0158 (6)	0.0111 (6)	0.0045 (5)	0.0098 (5)
N1	0.0163 (5)	0.0195 (5)	0.0175 (5)	0.0041 (4)	0.0023 (4)	0.0102 (4)
N2	0.0175 (5)	0.0163 (5)	0.0172 (5)	0.0053 (4)	0.0034 (4)	0.0098 (4)
N3	0.0161 (5)	0.0211 (5)	0.0165 (5)	0.0061 (4)	0.0026 (4)	0.0106 (4)
N4	0.0178 (5)	0.0163 (5)	0.0164 (5)	0.0059 (4)	0.0026 (4)	0.0081 (4)
N5	0.0183 (5)	0.0171 (5)	0.0163 (5)	0.0027 (4)	0.0019 (4)	0.0088 (4)
O1	0.0331 (5)	0.0208 (5)	0.0164 (5)	0.0081 (4)	0.0070 (4)	0.0077 (4)
O2	0.0335 (5)	0.0227 (5)	0.0237 (5)	0.0103 (4)	0.0056 (4)	0.0158 (4)
O11	0.0258 (5)	0.0171 (4)	0.0179 (5)	0.0038 (4)	0.0050 (4)	0.0095 (4)
O21	0.0334 (5)	0.0171 (4)	0.0150 (5)	0.0115 (4)	0.0060 (4)	0.0081 (4)

### Geometric parameters (Å, º)

**Table:**

C1—N3	1.3252 (16)	C21—C22	1.4063 (18)
C1—N1	1.3559 (16)	C21—N2	1.4111 (15)
C1—N5	1.4675 (16)	C22—O21	1.3588 (15)
C11—C16	1.3926 (17)	C22—C23	1.3949 (17)
C11—C12	1.4013 (18)	C23—C24	1.3872 (18)
C11—N4	1.4089 (16)	C23—H23	0.95
C12—O11	1.3608 (14)	C24—C25	1.3884 (19)
C12—C13	1.3911 (17)	C24—H24	0.95
C13—C14	1.3952 (18)	C25—C26	1.3869 (18)
C13—H13	0.95	C25—H25	0.95
C14—C15	1.3847 (19)	C26—H26	0.95
C14—H14	0.95	C27—O21	1.4319 (16)
C15—C16	1.3880 (18)	C27—H27A	0.98
C15—H15	0.95	C27—H27B	0.98
C16—H16	0.95	C27—H27C	0.98
C17—O11	1.4278 (15)	N1—N2	1.2897 (15)
C17—H17A	0.98	N3—N4	1.3005 (15)
C17—H17B	0.98	N4—H4	1.10 (3)
C17—H17C	0.98	N5—O2	1.2292 (14)
C21—C26	1.3953 (18)	N5—O1	1.2342 (15)
			
N3—C1—N1	134.09 (12)	O21—C22—C21	116.00 (11)
N3—C1—N5	112.67 (11)	C23—C22—C21	119.20 (11)
N1—C1—N5	113.09 (11)	C24—C23—C22	120.17 (12)
C16—C11—C12	120.02 (11)	C24—C23—H23	119.9
C16—C11—N4	122.24 (12)	C22—C23—H23	119.9
C12—C11—N4	117.68 (11)	C23—C24—C25	120.55 (12)
O11—C12—C13	125.15 (12)	C23—C24—H24	119.7
O11—C12—C11	115.31 (11)	C25—C24—H24	119.7
C13—C12—C11	119.54 (11)	C26—C25—C24	119.98 (12)
C12—C13—C14	119.96 (12)	C26—C25—H25	120
C12—C13—H13	120	C24—C25—H25	120
C14—C13—H13	120	C25—C26—C21	119.99 (12)
C15—C14—C13	120.36 (12)	C25—C26—H26	120
C15—C14—H14	119.8	C21—C26—H26	120
C13—C14—H14	119.8	O21—C27—H27A	109.5
C14—C15—C16	120.00 (11)	O21—C27—H27B	109.5
C14—C15—H15	120	H27A—C27—H27B	109.5
C16—C15—H15	120	O21—C27—H27C	109.5
C15—C16—C11	120.10 (12)	H27A—C27—H27C	109.5
C15—C16—H16	120	H27B—C27—H27C	109.5
C11—C16—H16	120	N2—N1—C1	114.18 (11)
O11—C17—H17A	109.5	N1—N2—C21	115.34 (10)
O11—C17—H17B	109.5	N4—N3—C1	117.69 (11)
H17A—C17—H17B	109.5	N3—N4—C11	117.09 (10)
O11—C17—H17C	109.5	N3—N4—H4	113.3 (17)
H17A—C17—H17C	109.5	C11—N4—H4	129.2 (17)
H17B—C17—H17C	109.5	O2—N5—O1	123.48 (11)
C26—C21—C22	120.09 (11)	O2—N5—C1	118.27 (11)
C26—C21—N2	123.72 (12)	O1—N5—C1	118.25 (11)
C22—C21—N2	116.18 (11)	C12—O11—C17	117.37 (10)
O21—C22—C23	124.80 (12)	C22—O21—C27	116.76 (10)
			
C16—C11—C12—O11	−178.36 (11)	C22—C21—C26—C25	0.55 (19)
N4—C11—C12—O11	4.19 (17)	N2—C21—C26—C25	179.30 (12)
C16—C11—C12—C13	1.18 (19)	N3—C1—N1—N2	−8.2 (2)
N4—C11—C12—C13	−176.26 (11)	N5—C1—N1—N2	176.64 (10)
O11—C12—C13—C14	178.79 (12)	C1—N1—N2—C21	178.01 (10)
C11—C12—C13—C14	−0.71 (19)	C26—C21—N2—N1	6.28 (17)
C12—C13—C14—C15	−0.8 (2)	C22—C21—N2—N1	−174.93 (11)
C13—C14—C15—C16	1.89 (19)	N1—C1—N3—N4	6.7 (2)
C14—C15—C16—C11	−1.41 (19)	N5—C1—N3—N4	−178.05 (10)
C12—C11—C16—C15	−0.12 (19)	C1—N3—N4—C11	−171.54 (10)
N4—C11—C16—C15	177.20 (11)	C16—C11—N4—N3	17.56 (18)
C26—C21—C22—O21	178.75 (11)	C12—C11—N4—N3	−165.06 (11)
N2—C21—C22—O21	−0.09 (16)	N3—C1—N5—O2	14.10 (16)
C26—C21—C22—C23	−1.06 (18)	N1—C1—N5—O2	−169.63 (10)
N2—C21—C22—C23	−179.90 (11)	N3—C1—N5—O1	−166.06 (11)
O21—C22—C23—C24	−179.23 (12)	N1—C1—N5—O1	10.20 (16)
C21—C22—C23—C24	0.57 (19)	C13—C12—O11—C17	−0.99 (18)
C22—C23—C24—C25	0.4 (2)	C11—C12—O11—C17	178.52 (10)
C23—C24—C25—C26	−1.0 (2)	C23—C22—O21—C27	−3.81 (18)
C24—C25—C26—C21	0.5 (2)	C21—C22—O21—C27	176.39 (11)

### Hydrogen-bond geometry (Å, º)

**Table:**

*D*—H···*A*	*D*—H	H···*A*	*D*···*A*	*D*—H···*A*
N4—H4···N2	1.10 (3)	1.73 (3)	2.6117 (15)	134 (3)
N4—H4···N1	1.10 (3)	2.42 (3)	2.8812 (16)	103 (2)
C17—H17*B*···O1^i^	0.98	2.47	3.3325 (16)	146
C27—H27*C*···O2^i^	0.98	2.65	3.3907 (17)	133

Symmetry code: (i) −*x*+1, −*y*+1, −*z*+1.
